# Polycarbonate Microchip Containing CuBTC-Monopol Monolith for Solid-Phase Extraction of Dyes

**DOI:** 10.1155/2020/8548927

**Published:** 2020-02-11

**Authors:** Eman Alzahrani

**Affiliations:** Chemistry Department, Faculty of Science, Taif University, Taif, Saudi Arabia

## Abstract

In the present study, preparation of CuBTC-monopol monoliths for use within the microchip solid phase extraction was undertaken through a 20-min UV lamp-assisted polymerization for 2,2-dimethoxy-2-phenyl acetophenone (DMPA), butyl methacrylate (BMA), and ethylene dimethacrylate (EDMA) alongside inclusion of the porogenic solvent system (1-propanol and methanol (1 : 1)). The resultant coating underwent coating using CuBTC nanocrystals in ethanolic solution of ethanolic solution of 1,3,5-benzenetricarboxylic acid (H_3_BTC, 10 mM) and 10 mM copper(II) acetate Cu(CH_3_COO)_2_. This paper reports enhanced extraction, characterization, and synthesis studies for porous CuBTC metal organic frameworks that are marked by different methods including SEM/EDAX analysis, atomic force microscopy (AFM), and Fourier-transform infrared spectroscopy (FT-IR). The evaluation of the microchip's performance was undertaken as sorbent through retrieval of six toxic dyes (anionic and cationic dyes). Various parameters (desorption and extraction step flow rates, volume of desorption solvent, volume of sample, and type of desorption solvent) were examined to optimize dye extraction using fabricated microchips. The result indicated that CuBTC-monopol monoliths were permeable with the ability of removing impurities and attained high toxic dye extraction recovery (83.4–99.9%). The assessment of reproducibility for chip-to-chip was undertaken by computing the relative standard deviations (RSDs) of the six dyes in extraction. The interbatch and intrabatch RSDs ranged between 3.8 and 6.9% and 2.3 and 4.8%. Such features showed that fabricated CuBTC-monopol monolithic disk polycarbonate microchips have the potential of extracting toxic dyes that could be utilized for treating wastewater.

## 1. Introduction

New porous components, for instance, metal-organic frameworks (MOFs), constitute an interesting category of crystalline components [1–4] that are based on the combination of metallic clusters or ions with complex organic connectors, forming extended sequenced networks [5–9]. Significant research attention has been accorded to MOFs because of their low density [10–12], adsorption capacity [13], chemical and thermal stabilities [14], large surface area [15], high porosity [16, 17], and large-sized pores [18]. Their three-dimensional (3D) crystalline structures are made from organic linkers, metallic clusters, or metallic ions [19–22]. A traditional technique for MOF synthesis is through solvothermal reaction that generated fine-particle powder [23–25].

For industrial application of such MOF powders, they must be transformed into structures that can be monoliths or particles [26]. MOF particles usually suffer from irregular shapes and widely distributed sizes that might lead to undesirable peak shapes, high column backpressure, and low column efficiency [27–29]. The most energy-efficient and economical structure for application within any given absorption system might be in monolith form as its structure is characterized by high mass transfer and low pressure reduction rates than other structures [30, 31]. Experimental studies examining the absorption processes of MOFs are usually conducted with its powder and structured MOF production, especially the industrial adsorption process monolithic structure is rare [32–34].

MOF-polymer composites that shape mixed-matrix monoliths for catalysis [35–37], separation [38–42], and gas storage [43–45] have already been examined. In the previous years, MOF use within the field of analytical chemistry has been increasing constantly [7, 46–48]. Nevertheless, such composite components are yet to be examined as SPE supports. The possible benefits of MOF-matrix SPE supports are as follows: (i) simple sorbent preparation allowing enrichment of targeted compounds and concurrently the exclusion of size within desorption phase of larger molecular sized compounds than the chosen MOF pore size, (ii) simple sorbent functionalization, merely through selection of proper organic connecters utilized within the synthesis of MOF, (iii) simple SPE automation process through flow-driven methods, avoiding high backpressure or tiny particles clogging the flow manifold tubing, and (iv) excellent flow-through characteristics, allowing SPE applications to use MOFs (independently regardless of their shape and crystal size) [44, 49–52].

The pattern in analysis towards microfluidic devices can be attributed to the ability of integrating several analytical processes into one microfluidic device, reduced reagent consumption, decrease in the time of analysis, and sample volume reduction [53–55]. A trend for using microfluidic devices on tasks including chemical modification, immunoassays, separation, and sample pretreatment exists [56–61]. This work is aimed at exploring the application of polycarbonate microchips with the CuBTC-monopol monolithic disk as dye extraction supports. HKUST-1 that constitutes one extensively examined MOF, experimentally and theoretically, is obtained from benzene-1,3,5-tricarboxylate (BTC) connecter molecules and Cu^2+^ cations, which contain paddle wheel structures [62–65]. In the present study, HKUST-1 is utilized as a MOF model for demonstrating in situ stratum-by-stratum self-assembly technique for grafting MOFs on poly(BuMA-*co*-EDMA) (butyl methacrylate-co-ethylene dimethacrylate) monolith surfaces. Notably, studies exploring in situ synthesized MOF development within the porous polymer monolith as the SPE stationary phase are nonexistent. The emerging CuBTC-poly(BuMA-*co*-EDMA) monolith underwent evaluation and was suggested as the stationary extraction phase of various dyes, which were congo red, fast green, methyl orange, methyl blue, bromophenol blue, and coomassi brilliant blue.

## 2. Experimental

### 2.1. Chemicals and Materials

All chemicals were bought from commercial sources and utilized without more purification. Ammonium acetate buffers, acetone, methanol, and isopropanol were bought from Fisher Scientific (Loughborough, UK). Ethanol, acetonitrile, and 1-propanol were bought Scientific Laboratory Supplies (Nottingham, UK). Congo red, methyl orange, fast green, bromophenol blue, coomassi brilliant blue, methyl blue, 1,3,5-benzenetricarboxylic acid (H_3_BTC), copper(II) acetate Cu(CH_3_COO)_2_, 2,2-dimethoxy-2-phenyl acetophenone 99% (DMPA), ethylene dimethacrylate 98% (EDMA), and butyl methacrylate 99% (BuMA) were bought from Sigma-Aldrich (Poole, UK).

### 2.2. Instrumentation

The FT-IR spectrum included PerkinElmer RXFTIR ×2 containing DRIFT attachment and diamond ATR (PerkinElmer-Buckinghamshire, UK). XRD was get using the D8-ADVANCE Bruker diffractometer (Coventry, UK). High-resolution atomic force microscopy (AFM) was utilized for testing morphological properties and to generate topological maps (Veeco-di Innova Model-2009-AFM-USA). The energy-dispersive analysis of X-ray spectroscopy (EDAX) and scanning electron microscope (SEM) equipment include Cambridge S360 from Cambridge Instruments (Cambridge, UK). UV-Vis spectrophotometers were purchased from Thermo-Scientific GENESIS 10S (Toronto, Canada). Microtight adapters were bought from Kinesis (Cambs, UK). Double/Bubble epoxy resin from Bondmaster Limited (London, UK). The ethylenetetrafluoroethylene (ETFE) tubing (1/16″ × 0.17 mm i.d.) was bought from Thames Restek Ltd. (Saunderton, UK). The pH meter was from Fisherman hydrus 300, Thermo Orion (Beverly, MA, USA). A baby bee syringe pump was acquired from Bioanalytical Systems Inc. (West Lafayette, USA). The UV lamp was bought from Spectronic Analytical Instruments (Leeds, UK). A sonicator was purchased from Ultrawave Sonicator U 300HD (Cardiff, UK), and 1 ml disposable plastic syringe was bought from Scientific Laboratory Supplies (Nottingham, UK).

### 2.3. Fabrication of the MOF-Polymer Monolith

Preparation of organic polymer-based monoliths in the plastic syringe in room temperature under UV irradiation was undertaken using photoinitiated free radical polymerization. The preparation of organic polymer-based monolith was undertaken based on descriptions presented in our study [66]. The polymerization mix featured the porogenic solvent system that constituted 1-propanol and methanol binary mixture (50 : 50), 2,2-dimethoxy-2-phenyl acetophenone (DMPA, the free radical photoinitiator), ethylene dimethacrylate (EDMA, crosslinker), and butyl methacrylate (BuMA, monovinyl monomer). Sonication was conducted on the mixture, which was then placed into the plastic syringe and heated using the UV lamp. The duration of exposure was 20 minutes, whereas the distance separating the plastic syringe and the UV lamp (365 nm at room temperature) was 5 cm. After monolithic materials have formed within the plastic syringe, methanol was used to wash the monolithic rod for 12 hours to eliminate the unreacted materials.

For preparation of CuBTC nanocrystals placed within the organic-based monolith mesoporosity, the monolithic materials that were prepared were immersed in a solution of ethanol containing Cu(CH_3_COO)_2_ (10 mM) for 120 minutes. After washing in ethanol, the monolithic material improved using Cu^2+^ was soaked within a BTC ethanolic solution (10 mM) for 120 minutes. Finally, the materials were subjected to washing and immersed in ethanol at 90°C for 12 hours, and then methanol added for preservation until used.

### 2.4. Fabrication of the Polycarbonate Microchip

The polycarbonate microchip's design contained two plates; base and top plate dimensions were 13.5 mm in width and length, and thickness of 2.8 mm. The top plate contained microchannels (depth 1 mm, width 1 mm, and length 4 mm) and two access holes (1.5 mm). The base plate had extraction chamber (2 mm depth and 6.5 mm width), which underwent milling using CNC (computer numerical control) milling machine. The monolithic organic disks were cut and placed in the extraction chamber [53, 67]. Epoxy resin was used for fixing the two plates, whereas ETFE tubing was placed into access holes drilled into microfluidic devices with epoxy resin. After the two plates were bonded, microchips were used for extracting dyes.

### 2.5. Characterisation of Fabrication Materials

The characterization of monolith's morphology was through SEM. Images were acquired using an increasing voltage of 20 kV alongside 100 pA probe current within high vacuum mode with SEM from Cambridge S360. Coating was undertaken on the samples using thin gold platinum layer (thickness about 2 nm) using a Nanotechnology Ltd. (Sandy, UK) SEMPREP 2 sputter coater. XRD with CuKα radiation in the 2 theta (2*θ*) range from 10° to 50°. Compositional analysis was examined with EDAX spectroscopy. Topological map (Veeco-di Innova Model-2009-AFM-USA) and morphological properties were tested using high-resolution AFM. Noncontacting mode tapping constituted the mode that was applied. Surface topology was accurately mapped by forwarding the AFM-raw data to the Origin-Lab version 6-USA program for accurate visualization of 3D surfaces for the sample being investigated. The FT-IR spectrum was gathered within the attenuated total reflectance (ATR) mode in ranges of 500–4000 cm^−1^.

### 2.6. Dye Extraction

The performance for microfluidic CuBTC-monopol monolith devices containing was investigated. The standardized dyes (congo red, methyl orange, fast green, bromophenol blue, commassi brilliant blue, and methyl blue) underwent dissolution in a buffer solution of ammonium acetate (10 mM, pH 9.3) and were utilized in extraction experiments. 100 *μ*L of ACN was used for activating the polymer-based monoliths within the microchip, which was then subjected to equilibration with using 100 *μ*L of 10 mM buffer solution of ammonium acetate (pH 9.3). The solution of the dye sample (100, 200, 300, or 400 *μ*L) was used on the monolithic microchips with flow rates of 5, 10, 15, or 20 *μ*L·min^−1^. Finally, extract portions (50, 100, 150, or 200 *μ*L) were eluted with eluents (acetone, acetonitrile, isopropanol, ethanol, or methanol) utilizing flow rates of 5, 10, 15, or 20 *μ*L·min^−1^ and collected into Eppendorf tubes and analyzed with the UV-Vis spectrophotomer. The reason for utilizing UV-Vis spectrophotomer was to examine the peak area of the preconcentrated dyes and comparing them against the peak levels of nonprocessed dye standardized solutions to compute the recovery of extraction. Calculation of the preconcentrated dyes' extraction efficiency was undertaken with the previous technique [1].

## 3. Results and Discussion

### 3.1. Fabrication of Metal-Organic Monolith

**Figure 1:**
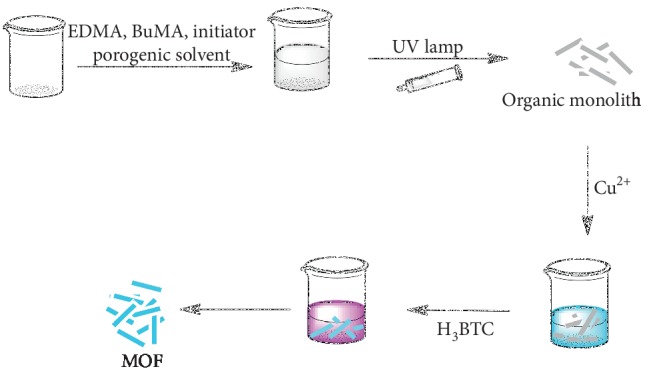
Steps of fabrication porous metal-organic (CuBTC-monopol) monolithic rod.

**Figure 2:**
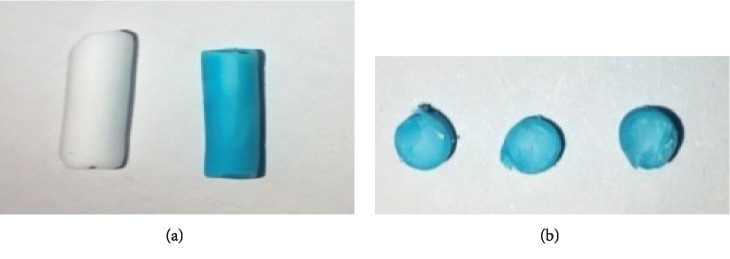
(a) The bare organic monolithic rod (left) and CuBTC-monopol monolith (right) and (b) cross-sectional view of CuBTC-monopol monolith (left, middle, and right).

This study sought to hierarchically fabricate porous metal-organic monoliths consisting of organic struts and metal clusters/ions, which integrate the advantages of lights aerogels and crystalline MOFs [68–71].  Figure 1 shows the major steps for fabricating CuBTC-monopol monolithic rods. The fabrication for the monoliths was premised on organic-based monolith fabrication followed by combination of Cu–BTC nanocrystals and organic monolithic components. The polymerized mix contained divinyl monomers (EDMA) and monovinyl (BuMA) within the existence of free radical initiators (DMPA) and porogenic solvents [72]. After putting the polymerized mixture into the plastic syringe, it was heated using the UV lamp, which led to the formation of polymers and later precipitation [72]. In the event the polymer-based monolith has bright white materials (satisfactory appearance), Figure 2(a), the washing of monolithic rods is undertaken. Washing of the soluble oligomer remnants within the pores, the unreacted monomeric material, and porogenic system solvents was undertaken using methanol for 12 hours to avert further polymerization reactions, since the crosslinker and the monomer have affinities of continuing the polymerization reactions [73].

The organic monolith formed underwent coating using CuBTC nanocrystals with ethanolic solution of H_3_BTC (10 mM) and ethanolic solution Cu(CH_3_COO)_2_ (10 mM). The resultant monolith (CuBTC-monopol) yields a homogeneous blue colored material showing homogenously distributed copper in the whole monolith, Figure 2(a). For generation of pure crystalline materials, the as-prepared monoliths in room temperature were soaked or washed in ethanol at 90°C for 12 hours. This sought to cause further reaction, increase porosity, and eliminate unreacted impurities to enhance the crystalline stage.  Figure 2(b) illustrates the cross section (bottom, middle, and top) of the MOF monolithic rods formed. The monolith's central and external parts have similar colors. The result proves the surface functionalization of organic based monoliths with equal CuBTC, and no recurrent treatment is needed to increase the CuBTC within the organic monoliths.

### 3.2. Physical Characteristics of CuBTC-Monopol Monolith

**Figure 3:**
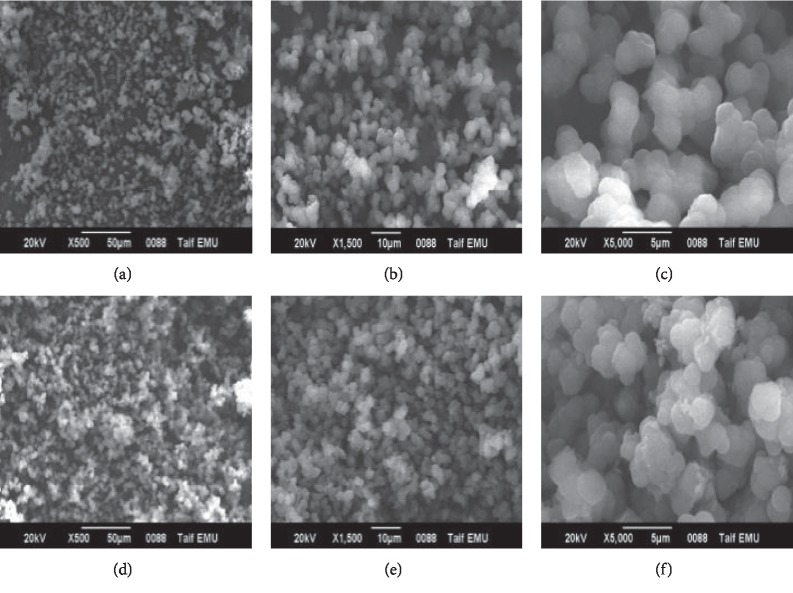
SEM images of the bare organic monolithic rod (a, b, c) and CuBTC-monopol monolith (d, e, f) using different magnifications.

**Figure 4:**
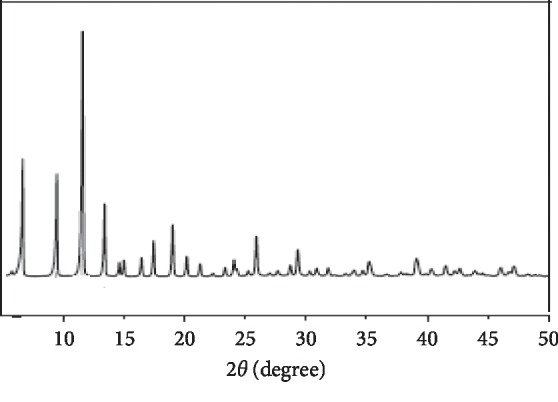
XRD pattern of CuBTC-monopol monolith.

After the drying phase, the monolith morphology was examined using SEM analysis. The analysis estimates the skeleton and through pore size, which would in turn determine the monolithic material's mechanical strength and hydrodynamic properties, whereas the approaches presented below are ideal for determining the through pore size distribution and exact size. The polymeric monoliths' morphologies after and prior to CuBTC-monopol monolith formation were examined through SEM analysis utilizing various magnifications, as can be seen in Figure 3. From the figure, it was found that all sample surfaces were porous, crack-free, smooth, and uniform structures. A suitable structure of the pore was illustrated clearly, showing homogenously interconnected open-pore macroporous networks. Besides, it was reported that the pore size reduced after CuBTC coating because the monolithic material was coated with metallic clusters/ions.  Figure 4 presents the XRD pattern of CuBTC-monopol monolith. This pattern is similar to the XRD pattern in the previous study [74] confirmed the formation of metal-organic frameworks.

**Figure 5:**
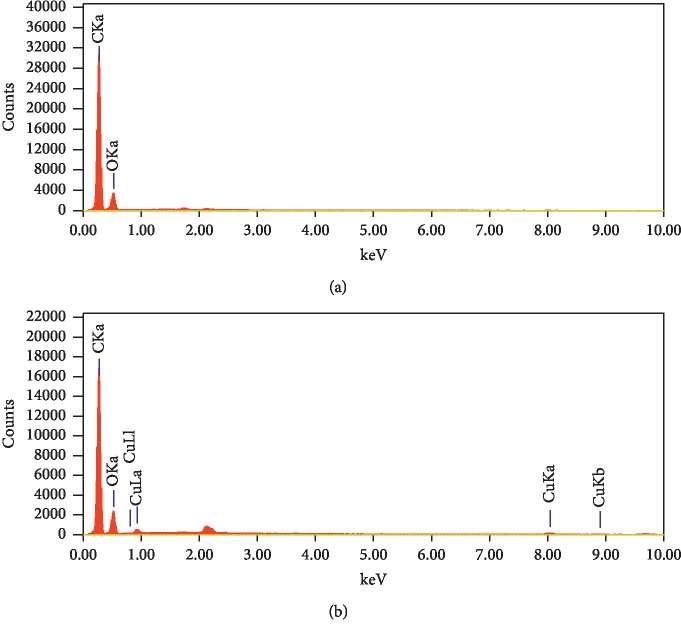
EDAX analysis of the bare organic monolithic rod (a) and CuBTC-monopol monolith (b).

EDAX analysis was used to further examine fabricated materials to determine the formed materials' composition. As Figure 5 illustrates that the EDXA spectra for bare organic monoliths revealed that the string peak for oxygen is 0.525 keV, whereas that for carbon is 0.227 keV. The CuBTC-monopol monolith's EDAX showed that, besides the oxygen and carbon element peaks, a peak of 8.040 keV which was linked to Cu was observed. The compositions of carbon (74.22%) and oxygen (25.78%) within the sample of organic-based monoliths are presented in Table 1. Notably, the composition of copper (1.88%) in CuBTC-monopol monoliths was found, thus confirming the modification to the organic monolith surface with a CuBTC layer.

**Figure 6:**
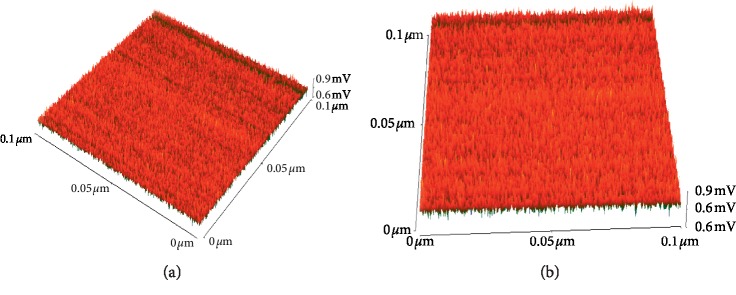
Three-dimensional AFM images recorded for bare organic monolithic material (a) and CuBTC-monopol monolith (b).

Notably, AFM was used for analyzing fabricated materials' surface properties, because the method could yield quantitative and qualitative information concerning the fabricated materials' topography.  Figure 6 illustrates the 3D fabricated materials' AFM images derived with the AFM method. As it could be observed clearly in Figure 6(a), the organic-based monolith's flat surface was uniform and smooth, with an optimum surface height of about 4.1 *μ*m. In Figure 6(b), the 3D CuBTC-monopol monolith AFM image revealed that the surface's maximum height rose to 6.4 *μ*m, thus proving that organic monoliths had been coated with a layer of CuBTC.

**Figure 7:**
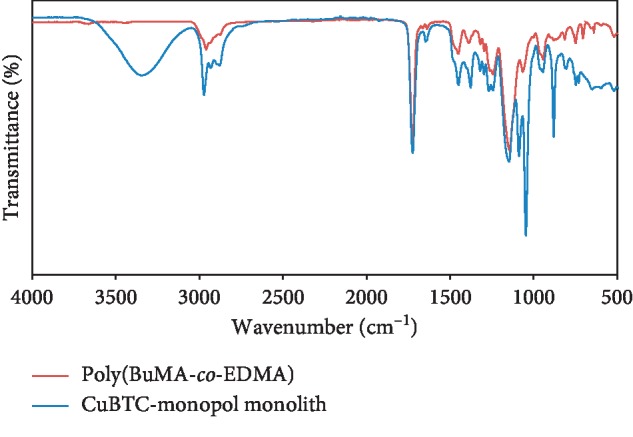
FT-IR spectra of bare organic monolithic material and CuBTC-monopol monolith.

The prepared materials' composition was characterized using the FT-IR spectrometer.  Figure 7 presents the FT-IR spectrums; the two spectra are identical with the following string peaks, cm^−1^: 1273–1000 C − O − C [75] vibration within ester groups, 2875–2987 CH stretching within CH_2_ and CH_3_ groups, 1732 C=O stretching, and the peak for carbonyl is 1675 cm^−1^. Notably, the sole variation on the copolymer spectra pertains to the sharp centered band at 1637 cm^−1^, connected to carbonyl from ester groups originating from EDMA/BuMA alongside other O − H peak at 3438 cm^−1^ [76].

### 3.3. Dye Extraction

**Figure 8:**
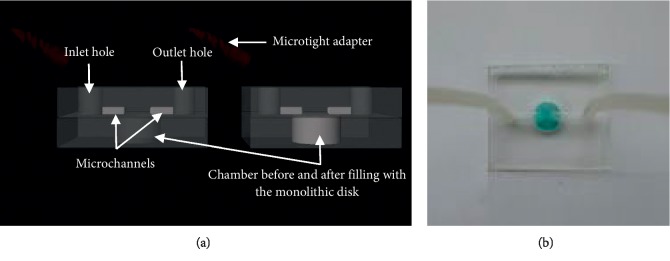
Schematic diagram and a photograph of the polycarbonate microchip. The top and base plates were 13.5 mm in length and width and the thickness was 2.8 mm. The top plate contained two microchannels (length 4 mm, width 1 mm, and depth 1 mm) and two access holes (1.5 mm) for attachment of ETFE tubing while the base plate contained the extraction chamber (6.5 mm width and 2 mm depth).

This study sought to fabricate polycarbonate microchips containing effective dye extraction sorbents (cationic and anionic dyes). The microchip's photograph and schematic diagram, which was utilized for extracting dyes, is presented in Figure 8. The polycarbonate microchip's design featured two plates, with the top plate having microchannels (depth 1 mm, width 1 mm, and length 4 mm) alongside access holes (1.5 mm) for ETFE tubing attachment, whereas the base plates contained an extraction chamber (2 mm depth and 6.5 mm width). The CuBTC-monopol monolith disk's diameter (2 mm) was modified using scalpel blades. Epoxy resin was used for fixing the tubing of ETFE within the access holes. The microtight adapter was employed for connecting the ETFE tubing to plastic syringes that were attached to a syringe pump.

This study sought to locate the ideal dye extraction performance. Various dyes were examined in the study including congo red, methyl orange, fast green, bromophenol, coomassi brilliant blue, and methyl blue. As the first experiment, 100 *μ*L of ACN was used for activating the polymer-based monoliths within the microchip, which was then subjected to equilibration using 100 *μ*L of 10 mM buffer solution of ammonium acetate (pH 9.3). The solution of the dye sample (300 *μ*L) was injected into the polycarbonate microchip using a syringe pump at a flow rate of 10 *μ*L·min^−1^, and when the solution of the sample was loaded via extraction monolith, the sorbent was washed using ACN and rinsed to eliminate impurities and keep the analytes. Finally, the preconcentrated dyes were eluted with desorption solvent (150 *μ*L) with a flow rate of 5 *μ*L·min^−1^.

#### 3.3.1. Selection of the Desorption Solvent

**Figure 9:**
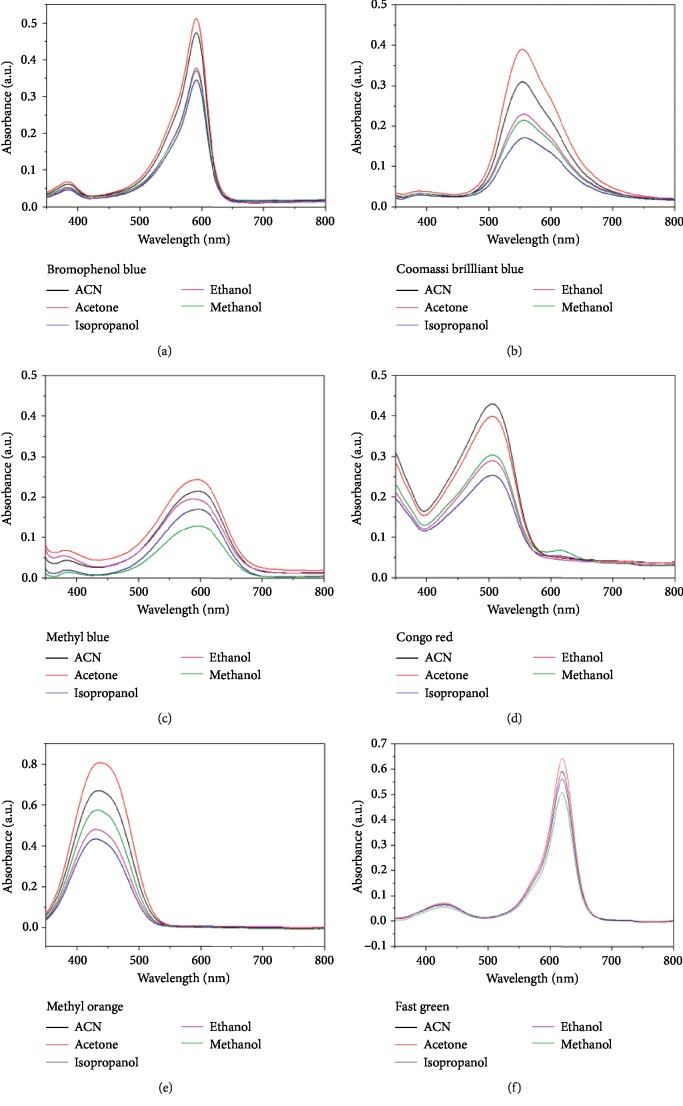
UV-Vis spectra of the preconcentrated dyes using different desorption solvents (acetone, acetonitrile, isopropanol, ethanol, or methanol).

**Figure 10:**
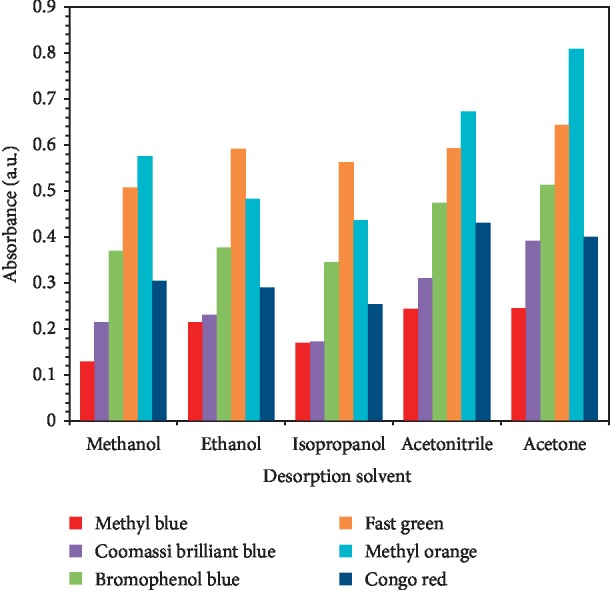
Effect of desorption solvent selection on the desorption of the dyes from the CuBTC-monopol monolithic disk inside the polycarbonate microchip. Conditions: sample volume, 300 *μ*L; sample flow rate, 10 *μ*L·min^−1^; dye concentration, 60 mg·L^−1^; desorption solvent volume, 150 *μ*L; flow rate, 10 *μ*L·min^−1^.

The effect from various desorption solvents including acetone, acetonitrile, isopropanol, ethanol, and methanol on the CuBTC-monopol monolithic disk analyte desorption was examined to obtain the suitable conditions of desorption for polycarbonate microchip dyes. The solution of the dye sample was pumped into the polycarbonate microchip and ACN used for washing the sorbents, and finally the preconcentrated dyes were eluted with desorption solvent and gathered into Eppendorf tube. The UV-Vis spectrophotometry was used for measuring the preconcentrated dye peak areas. The preconcentrated dyes' UV-Vis spectra using various desorption solvents are presented in Figure 9. From Figure 10, it can be seen that all examined solvents were used for desorption of various dyes; nevertheless, it was reported that application of acetone as desorption solvents could accelerate extraction of various sorbent dyes. Thus, in all examined dyes, the desorption solvent was acetone.

#### 3.3.2. Study of the Extraction Parameters

Desorption steps and extraction flow rates, desorption solvent volume, and sample volume are important parameters for designing SPE procedures conducted using flow-based methods operating in nonequilibrium conditions. All such parameters were examined to increase the dye extraction using fabricated polycarbonate microchips.

**Figure 11:**
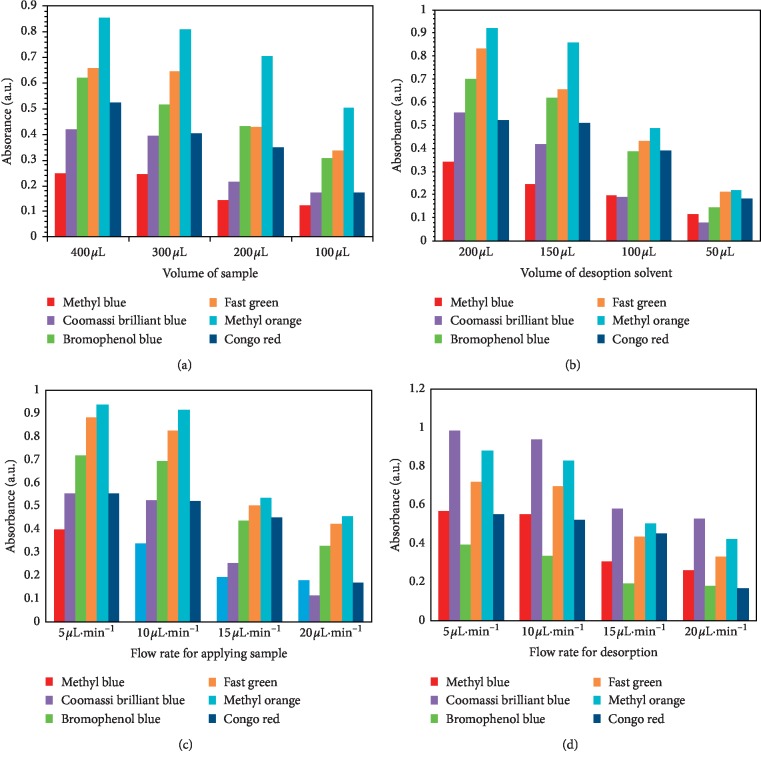
Effect of volume of dye sample solution on the extraction efficiency of dyes (a), volume of acetone (b), flow rate of applying sample (c), and flow rate for desorption solvent (d). Conditions: sample volume, 400 *μ*L; flow rate, 10 *μ*L·min^−1^; dye concentration, 60 mg·L^−1^; desorption solvent (acetone) volume, 200 *μ*L; flow rate, 5 *μ*L·min^−1^.

The dye sample solution's volume 100, 200, 300, and 400 *μ*L was another optimized parameter. The sample volume effect on dye preconcentration is shown in Figure 11(a). Under the chosen experimental conditions, it could be observed that the sample volume of 400 *μ*L indicated the suitable result. The extracted analyte quantity increased when the volume of the sample was increased from 100 *μ*L to 400 *μ*L. In view of this, the dye solution's volume at 400 *μ*L was chosen for future experiments.

The effects of desorption acetone volume on extracted dye elution from CuBTC-monopol monolithic disk is illustrated in Figure 11(b). The volume of the desorption solvent from 50 *μ*L to 200 *μ*L to decrease the consumption of solvent within desorption phase, while ensuring proper desorption of retained sorbent analytes. The method's performance increased when the volume of the desorption solvent increased. In view of this, the volume of desorption solvent (200 *μ*L) was chosen for more experiments.

The flow rate of the extracted sample was investigated within the range of 5 *μ*L·min^−1^–20 *μ*L·min^−1^. In higher rates of flow, analyte extraction decreases slightly as illustrated in Figure 10(c). The increase in the flow rate of the sample reduces the time of contact between CuBTC-monopol monolith and dyes, reducing the mass transfer and thus the extracted dye quantity. In the compromise involving high extraction throughputs and high extraction efficiencies, the extraction phase flow rate of 10 *μ*L·min^−1^ was used for additional experiments.

The effects of desorption solvent's flow rates on retained analytes desorption from CuBTC-monopol monoliths are shown in Figure 11(d). The flow rate effect on desorption phase follows a trend that resembles that for the loading phase. An increase in the flow rates of desorption solvent decreases the time of contact between the retained dye sorbents and desorption solvent; this also decreases the solvent action on the process of desorption. The effects for desorption solvent's flow rate were investigated from 5 *μ*L·min^−1^–20 *μ*L·min^−1^. The greatest rate of flow, which enabled maximum effectiveness on desorption phase, was 5 *μ*L·min^−1^, being used for experiments.

#### 3.3.3. Real Sample Analysis

**Figure 12:**
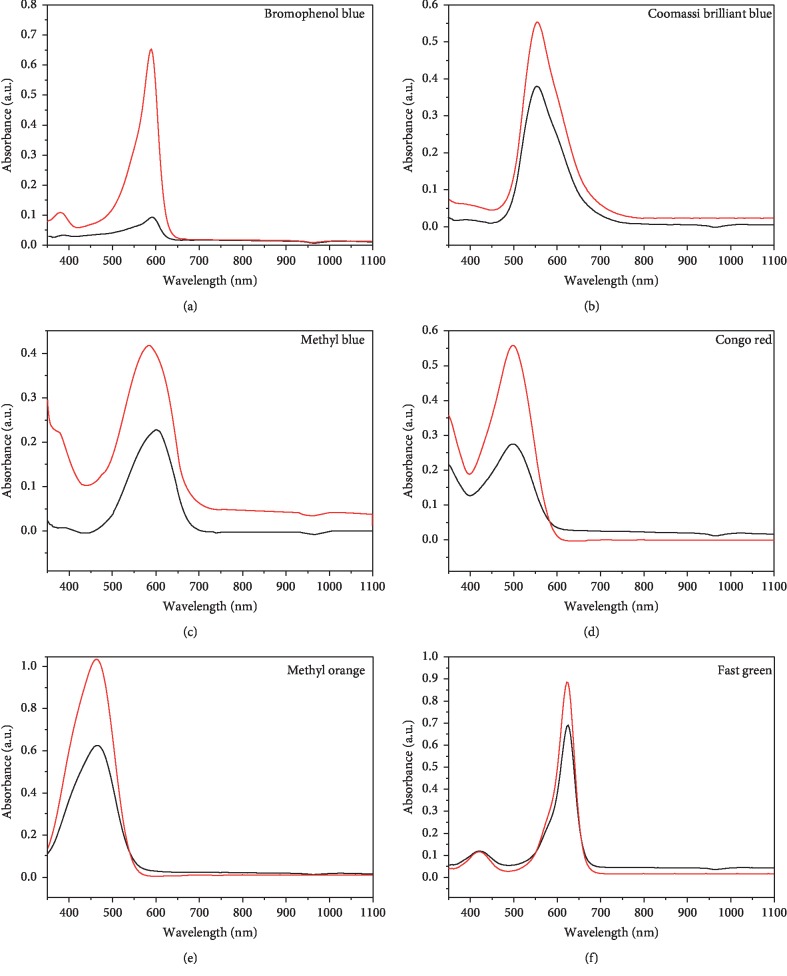
HPLC chromatograms of the direct injection of a standard spiked with the analytes (black line) and after preconcentration using the fabricated polycarbonate microchip containing CuBTC-monopol monolithic disk (red line).

To investigate the applicable nature of polycarbonate microchips containing CuBTC-monopol monolithic disks for dye SPE, various polluted samples of groundwater were subjected to analysis. Samples of groundwater were gathered from reservoirs of water. Dyes were used for spiking the samples, and a recovery study was conducted by having the samples spiked. Calculation of analyte recoveries was undertaken as the dye concentration ratio measured within the spiked sample and within pure spiked water with similar level of concentration. The chromatograph for samples of polluted groundwater spiked with dye after and before preconcentration using fabricated microchips is shown in Figure 12, and Table 2 shows the results obtained. Following spiking, the recoveries obtained ranged between 78.2% and 92.5% when using unmodified organic monolith, while the recoveries obtained ranged between 83.4% and 99.9% for all analyzed samples. Such results confirm polycarbonate microchip containing CuBTC-monopol monolith suitability for analysis of toxic dyes samples.

#### 3.3.4. Limit of Detection and Quantification

During calibration, five samples were used with different standard solutions for the analyzed dyes. For each sample, three tests were done and calibration curve calculated using the least-squares method. The curve generated was concentration against peak area with correlation coefficient. The curves generated were linear where the limit of detection (LOD) and limit of quantitation (LOQ) values were determined. The calculations were based on standard deviation of the detector response (*σ*) and the slope (*S*) of the curve, and the validation of analytical procedures is as demonstrated in the following expressions:

(1) $$\begin{array}{c} \text{LOD}=\frac{3\sigma }{S},\\ \text{LOQ}=\frac{10\sigma }{S}. \end{array}$$

The value of response *σ* is the standard deviation of the *y* intercept of the regression line determined from the plotted calibration curves. The analysis will only take a proportion of efficiency during an extraction. *S* Denotes the slope of the regression corrected for the SPE recovery value.

During the experiment, six dyes were analyzed including bromophenol blue, coomassi brilliant blue, methyl blue, congo red, methyl orange, and fast green. The HPLC values for these dyes were recorded and corrected for SPE recovery values which were then used to determine the LOD and LOQ calculation for the calibration curves. The values of LODs and LOQs were recorded as shown in Table 3.

#### 3.3.5. Reproducibility of the Fabricated Device

The CuBTC-monopol monolith fabrication's reproducibility constitutes an important aspect in the procedure's practicality and effectiveness, since CuBTC-monopol monolith fabrication constitutes a multiphase process. Additionally, surface modification reproducibility for CuBTC layer monoliths constitutes a crucial requirement for recognition of the preparation method used, particularly within industrial laboratories. Besides, reusability is one important aspect of using polycarbonate microchips, because fabrication costs are high. The fabrication technique's reproducibility of three monoliths fabricated from three polymerization mixes have a similar composition.

The monolith fabrication's reproducibility was evaluated by verifying the morphologies that SEM found for three separate batches of fabricated CuBTC-monopol monoliths. It was reported that visible differences did not exist within the prepared monolith's morphology. Nevertheless, CuBTC-monopol monolith within polycarbonate microchips involve cutting monolithic organic rods using scalpel blades and putting the monolithic CuBTC-monopol disks in polycarbonate microchip's base plate extraction chamber prior to bonding base and top plates using epoxy resin that can cause differences in prepared monolith's size, which in return might cause variability for dye extraction. It is proposed that such variability could be reduced through automated preparatory procedure.

The performance's reproducibility of fabricated devices was examined by computing the extraction efficiency's relative standard deviation (RSD) for six samples of dyes. Extraction efficiency's intrabatch RSDs of preconcentrated dyes using the same microchip (4 runs) as well as using various microchips (*n* = 3) are shown in Table 3. The obtained results from the investigation indicated that the dye preconcentration procedure was reproducible because acceptable run-to-run reproducibility was attained using RSD values ranging from 2.3 to 4.8%, while interbatch RSDs ranged between 3.8 and 6.9% for chip-to-chip reproducibility.

The capacity of size exclusion for developed SPE support offers more advantages for analyzing chemicals; for instance, enhanced selectivity for analysis of chemicals through chromatographic methods, sample matrix simplification before injection to analytical instrumentation, and increased selectivity of tiny molecule analysis. The major pitfalls regarding the CuBTC-monopol monolith use as SPE sorbents pertain to the limited stability for CuBTC-monopol monolith within acidic media and limited commercial availability of CuBTC-monopol monoliths. Nevertheless, several CuBTC-monopol monoliths could easily be synthesized from commercially available and cheap precursors and have stability towards experimental conditions utilized in different SPE applications. The advantages of the former, alongside their facile automation, as well as versatile and simple preparation provide CuBTC-monopol monolith with a variety of possibilities for analytically preparing samples.

## 4. Conclusion

The preparation of samples is regarded as barrier in the chemical analysis system. This study sought to design a CuBTC-monopol monolith dye-extraction microchip. The monolithic CuBTC-monopol material constitutes high-porosity materials with huge potential for application as toxic dye preconcentration sorbent. The analysis of monolithic CuBTC-monopol material was undertaken using various methods, including SEM/EDAX analysis, AFM analysis, and FT-IR spectroscopy. In the current work, successful designing of novel microfluidic devices that contain crack-free organic monoliths was modified using CuBTC-monopol groups to decrease reagents and analyte volume. The fabricated polycarbonate microchips were evaluated for dye extraction. Different parameters, including desorption steps, extraction flow rates, volume of desorption solvent, sample volume, and type of desorption solvent, were examined to optimize dye extraction using fabricated polycarbonate microchips. The results indicate that modified monoliths yielded reproducible extraction efficiency for various dyes and had stability over time and low flow resistances. Work is underway to create fully integrated preconcentration devices of various toxic dyes derived from actual samples as well as photodegradation of such dyes.

## Figures and Tables

**Table 1:** Elemental composition of the fabricated materials determined by EDAX analysis.

Sample	Element	keV	Mass (%)	Total
Organic-based monolith	C	0.277	74.22	100.00
	O	0.525	25.78	

CuBTC-monopol monolith	C	0.277	67.27	100.00
	O	0.525	30.85	
	Cu	8.040	1.88	

**Table 2:** Extraction efficiency percent of the investigated dyes from potentially polluted groundwater samples using organic-based monolith and CuBTC-monopol monolith.

Dyes	Type of dye	Wavelength (nm)	Extraction efficiency (%) using organic-based monolith	Extraction efficiency (%) using CuBTC-monopol monolith
Bromophenol blue	Basic	590	92.5	99.9
Coomassi brilliant blue		553	81.7	87.1
Methyl blue		593	86.3	91.5

Congo red	Acidic	497	88.2	93.6
Methyl orange		469	84.8	90.2
Fast green		623	78.2	83.4

**Table 3:** Limit of detection (LOD), limit of quantitation (LOQ) values, linearity of the analytical detection for the different dyes, and the intrachip (run-to-run) reproducibility and inter-chip (chip-to-chip) reproducibility of the polycarbonate microchip containing the CuBTC-monopol monolithic disk.

Dyes	Regression coefficient *R*^2^ (*n* = 3)	LOD (*S*/*N* = 3) (mg·L^−1^)	LOQ (*S*/*N* = 10) (mg·L^−1^)	Intra-chip (*n* = 4)	Inter-chip (*n* = 3)
Bromophenol blue	0.9971	31	102	2.3	3.8
Coomassi brilliant blue	0.9962	11	38	3.7	4.6
Methyl blue	0.9957	20	32	4.5	4.9
Congo red	0.9990	19	25	3.1	5.1
Methyl orange	0.9980	17	23	4.6	5.4
Fast green	0.9909	29	68	4.8	6.9

## Data Availability

The data used to support the findings of this study are included within the article.
